# Packaging signals in single-stranded RNA viruses: nature’s alternative to a purely electrostatic assembly mechanism

**DOI:** 10.1007/s10867-013-9313-0

**Published:** 2013-04-12

**Authors:** Peter G. Stockley, Reidun Twarock, Saskia E. Bakker, Amy M. Barker, Alexander Borodavka, Eric Dykeman, Robert J. Ford, Arwen R. Pearson, Simon E. V. Phillips, Neil A. Ranson, Roman Tuma

**Affiliations:** 1 Astbury Centre for Structural Molecular Biology, University of Leeds, Leeds, LS2 9JT UK; 2 Departments of Biology and Mathematics & York Centre for Complex Systems Analysis, University of York, York, YO10 5DD UK; 3 Research Complex at Harwell, Rutherford Appleton Laboratory, Harwell Oxford, Didcot, Oxon OX11 0FA UK; 4 Present Address: Centre for Virus Research, University of Glasgow, Glasgow, G11 5JR UK

**Keywords:** Virus assembly mechanism, RNA–protein interactions, Packaging signals, Fluorescence spectroscopy, Assembly models

## Abstract

The formation of a protective protein container is an essential step in the life-cycle of most viruses. In the case of single-stranded (ss)RNA viruses, this step occurs in parallel with genome packaging in a co-assembly process. Previously, it had been thought that this process can be explained entirely by electrostatics. Inspired by recent single-molecule fluorescence experiments that recapitulate the RNA packaging specificity seen in vivo for two model viruses, we present an alternative theory, which recognizes the important cooperative roles played by RNA–coat protein interactions, at sites we have termed packaging signals. The hypothesis is that multiple copies of packaging signals, repeated according to capsid symmetry, aid formation of the required capsid protein conformers at defined positions, resulting in significantly enhanced assembly efficiency. The precise mechanistic roles of packaging signal interactions may vary between viruses, as we have demonstrated for MS2 and STNV. We quantify the impact of packaging signals on capsid assembly efficiency using a dodecahedral model system, showing that heterogeneous affinity distributions of packaging signals for capsid protein out-compete those of homogeneous affinities. These insights pave the way to a new anti-viral therapy, reducing capsid assembly efficiency by targeting of the vital roles of the packaging signals, and opens up new avenues for the efficient construction of protein nanocontainers in bionanotechnology.

## Introduction

Viruses are major pathogens in all kingdoms of life. Single-stranded (ss)RNA viruses make up a significant fraction of these pathogens with detrimental impacts on human health. Their control via vaccination will only ever be possible for a limited subset of examples, so innovative routes to antiviral therapy are urgently required. Virion formation and uncoating are highly cooperative aspects of the viral lifecycle that are potential drug targets, but these have largely not been exploited thus far. One reason for this, with respect to assembly, is the apparent lack of specificity for RNA in in vitro assays of spontaneous co-assembly to form virus-like particles. Many viral coat proteins (CPs) also seem able to assemble correctly in the absence of RNA. The lack of sequence specificity has been interpreted to mean that assembly is driven largely by electrostatics [1–4]. RNAs carry a large amount of negative charge that can be neutralized by the positively charged domains or surfaces seen on many viral CPs. However, these in vitro results do not reflect the apparent specificity of genome encapsidation seen in vivo [5], where there is a clear biological imperative to (1) form virions packed with cognate genomes and not cellular RNAs; and to (2) complete the assembly process efficiently before the host defense mechanisms can clear the infection. We review here our recent data that suggest that while electrostatics clearly plays an important role in ssRNA virus capsid assembly, it overlooks the vital cooperative roles by which the genomic RNA facilitates efficient encapsidation in an environment in which capsid protein concentrations are much lower than in most in vitro studies. Thus the genome confers a distinct evolutionary advantage to assembly of these pathogens, as well as encoding their gene products. The deeper understanding of these mechanisms provided by our research paves the way for novel antiviral strategies, targeting these additional roles of the genome in capsid formation.

## In vitro assembly assays demonstrating packaging specificity

**Fig. 1:**
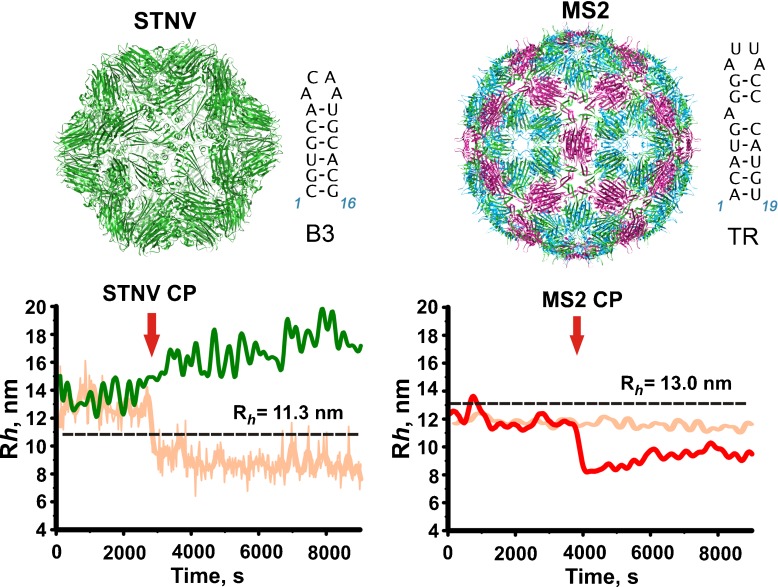
Demonstration of RNA packaging specificity for two model viruses. The *top row* shows external views of the capsids of STNV and bacteriophage MS2 based on their PDB entries (3 S4G & 1AQ3, respectively). These are examples of *T* = 1 and *T* = 3 capsids. Alongside are the sequences and secondary structures of their known packaging signals, B3 & TR, respectively [13, 18]. The *bottom row* shows the results of single-molecule fluorescence correlation spectroscopy (smFCS) assembly assays for both viruses. The hydrodynamic radii (R_*h*_), derived from the FCS curves, are plotted for both genomic RNAs (~1 nM) before and after (*red arrow*) the addition of sufficient coat protein subunits to allow assembly of completed capsids around each RNA. The protein-free RNAs show the presence of multiple conformers in equilibrium, most of which are too large to fit within their respective capsids (the *dashed black lines* and R_*h*_ values are for the external radii of the respective capsids). On addition of the cognate, and only the cognate, CP subunits there is a sudden collapse in the hydrodynamic radius of the resulting RNA-CP complexes. Electrostatic models of assembly would not predict this specificity or the collapse. Electron micrographs of the assembly reactions at the end of the FCS measurement show that the cognate reactions have produced the expected capsids in high yield [6]

We have recently established single-molecule fluorescence correlation spectroscopy (smFCS) assays to monitor the fates of dye-labeled CPs or RNAs during in vitro reassembly at the low CP concentrations typical of in vivo scenarios [6]. These allow the conformations of genomic RNAs before, during, and after encapsidation to be followed by direct estimation of their hydrodynamic radii (R_*h*_). The assays are sensitive in the nanomolar concentrations range (~1 nM for RNA & ~100 nM for CP), which are much more reflective of early concentrations within infected cells than most in vitro assays where CP concentrations of ≥10 $\upmu $M are common. Working with two model viruses, satellite tobacco necrosis virus (STNV) and bacteriophage MS2 (Fig. 1), we were able to show that labeled proteins and capsids had R_*h*_s derived from smFCS similar to those determined by other techniques, such as X-ray crystallography and mass spectrometry. Furthermore, the technique showed good discrimination between the starting materials (dis-assembled RNAs and CPs) and the end products (capsids) in in vitro reassembly reactions.

The most interesting information from these assays comes from dye-labeled genomic RNAs to which a full complement of CPs is added, allowing each RNA to form a capsid [6]. Initially in the absence of CPs, the genomic RNAs, and sub-fragments of the MS2 RNA (not shown), exhibit a broad range of R_*h*_ values, consistent with an ensemble of differing conformers in equilibrium (Fig. 1). This is consistent with the necessity of these RNAs playing multiple roles during the viral lifecycle, each of which requires a different conformational state. Most of the conformers seen in the absence of CP are too large to fit within the confines of their respective capsids. Upon addition of the cognate CPs there is a sudden collapse by up to 20% in R_*h*_ values that makes the resultant complex smaller than the diameter of the respective capsids. This event is followed by a slower recovery in R_*h*_ values that then plateau close to the expected values for intact capsids. The collapse only occurs with cognate CPs and RNAs. Non-cognate viral RNAs are equivalent to non-cognate cellular RNAs in these assays. Electron microscopy shows that the cognate reactions produce the expected capsids with high yield and fidelity. Non-cognate reactions also appear to stimulate assembly under these conditions, but do so highly inefficiently and produce a majority of misshapen and aggregated species with very few structures equivalent to a well-formed capsid. The collapse is a consequence of multiple CP-RNA and CP-CP contacts, since an MS2 CP mutant (W82R) [7] that binds RNA normally but cannot make the protein–protein contacts required for capsid formation does not induce collapse. It is also not CP concentration-dependent above a threshold value, as the amplitude of the collapse is constant. EMs of the MS2 reaction just after the collapse show partially formed capsid shells of the correct size and symmetry, implying that the complex forms in an ordered way and is not simply aggregated material that subsequently rearranges. The recovery stage is dependent on CP concentration as expected for recruitment of additional CP subunits to complete capsid formation. We interpret these data to mean that within each genome there are specific CP binding sites that are arranged in three dimensions to facilitate the CP-CP contacts seen in the capsid. The binding energy of these CP-RNA complexes is used to overcome the entropic costs associated with RNA confinement during assembly. We term the RNA sites involved in these contacts Packaging Signals (PSs). Some animal viruses, e.g., polio, are known to package predominantly nascent genomic transcripts as they emerge from the RNA-dependent RNA polymerase, rather than co-assembling with a fully formed RNA as in these experiments. However, in such cases the genomic RNAs still need to be confined and so a variant of the PS idea may still explain this behavior. In those cases, the PSs may only fold correctly for CP binding on the nascent transcript.

## The packaging signal hypothesis

**Fig. 2:**
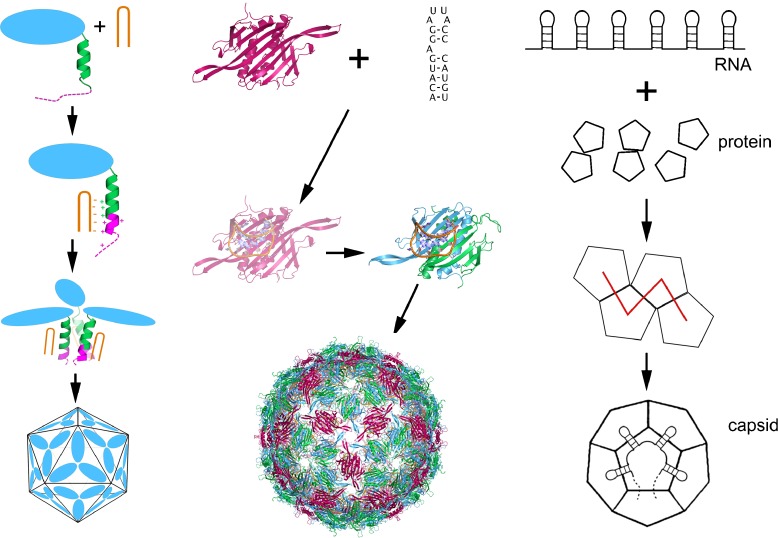
The effects of packaging signals. The *left-hand panel* shows the inferred assembly mechanism for STNV based on an X-ray crystal structure of a virus-like particle containing multiple copies of its packaging signal RNA and in vitro assembly assays [14]. The STNV CP in solution is monomeric and does not assemble in the absence of RNA. In the presence of the PS RNA, it assembles rapidly and the VLP structure shows that an additional section of the polypeptide chain towards the N-terminus becomes more ordered, extending the existing alpha helix by four residues. Helices from three adjacent CP subunits meet at the capsid three-fold axes. The additional amino acids being ordered contain several that are positively charged, suggesting that RNA binding overcomes an electrostatic barrier to assembly within the CP. This is the opposite to many views of assembly in this class of virus. The *middle panel* shows our current assembly model for MS2, which is nucleated by binding of its high affinity PS, the TR stem-loop, and proceeds by recruitment of further dimers, mediated via contacts with the other PSs. In this case, the CP is a dimer that is symmetrical in the absence of RNA but adopts the asymmetric conformer when bound to a PS. Both CP conformers are required to assemble the *T* = 3 shell. The *right-hand panel* shows a cartoon of the dodecahedral model system that we have used to analyze the principles of these co-assembly scenarios. In this case, the 12 pentagonal building blocks represent the units of assembly (capsomeres, here pentagons), and interact with the 12 packaging signals on a hypothetical RNA. PS-capsomere contacts are assumed to take place at the centers of the building blocks. In the fully assembled capsid, all PSs are bound to capsomeres, and the specific PS-capsomere pairing across the capsid defines the organization of the RNA in proximity to the dodecahedral capsid surface

The results described above suggest that there should be multiple PSs in the genomic sequences that play important functional roles via contacts with CP at defined positions in the capsid. For example, we have shown previously that contacts between characteristic stem-loop motifs in the MS2 sequence and CP subunits result in a conformational change of the symmetric, RNA-free CP dimer [7–11], the prevalent conformer in solution, to the asymmetric form required at 60 of the 90 dimer positions in the fully assembled capsid (Fig. 2a). Via such contacts, virus assembly is significantly accelerated from the order of days in the RNA-free case, to minutes if genomic RNAs or multiple copies of fragments encompassing PSs are present. This suggests a scenario of capsid assembly that we previously termed the dimer switching model (DSM) of assembly [12], in which the genomic RNA is organized in proximity of the capsid in such a way that it meets every asymmetric dimer, extending a stem-loop contact (PS) to CP as it does so, hence enabling the required conformer switch at defined positions in the assembling capsid.

Similarly, we have shown that contacts between RNA stem-loops in the STNV genome sequence and CPs at the three-fold axes of symmetry of the *T* = 1 capsid help overcome an electrostatic barrier between the CP monomers that prevents assembly in the absence of RNA (Fig. 2b) [13, 14]. Both scenarios are incarnations of the same co-assembly principle, that we call the *Packaging Signal Hypothesis*: Multiple copies of PSs fulfil the same function, albeit different ones in different viruses, at defined positions, repeated according to capsid symmetry, aiding formation of the correct/required CP conformers and thus resulting in significantly enhanced assembly efficiency.

These ideas follow on from proposals based on the X-ray structure of satellite tobacco mosaic virus (STMV), where up to 80% of the genomic RNA is ordered and has been modeled as a series of stem-loops positioned along the particle two-fold axes [15]. Further modeling based on a folded genomic RNA constrained to form 30 such stem-loops and chemical footprinting [16], has allowed a full three-dimensional model of the RNA to be built [17]. Unfortunately, in this case, it has not been possible to demonstrate the functional roles of these putative packaging signals directly. In all these cases, the implication is that genomic RNAs fulfil multiple functions, including formation of repeating motifs for interaction with their coat proteins in the virion, as well as encoding of the viral proteins and gene and replicative control elements.

## The nature of the packaging signals

High-affinity PSs have been identified in many viral systems, and their roles in initiating capsid assembly and conferring packaging selectivity have been discussed. In MS2, there is a single copy, high-affinity, stem-loop CP-binding site (TR) [18] that plays multiple roles in the viral lifecycle. It serves as an operator for a CP-induced translational repression of the replicase gene, and it is also believed to act as the point of assembly nucleation. In vitro reassembly assays show that flanking sequences also contribute to assembly efficiency [19–21] and there is genetic evidence that there are likely additional PSs throughout the genome [22]. However, the existence of multiple packaging signals, potentially with medium to low affinity for CP, as suggested by the above model, has previously been overlooked. This is perhaps due to the fact that PS motifs vary in their primary structures and reveal characteristic motifs only in the context of their secondary structures. We used clues from RNA SELEX [23], a method providing insights into relative affinities of RNA fragments to CP, to establish the existence of multiple PS consistent with the PS hypothesis in a number of viral systems.

For example, STNV *T* = 1 capsids in vivo package a genomic RNA consisting only of mRNA for the CP. There are three known viral strains. Unusually in this class of virus, our recombinant mRNA in *E. coli* cells is expressed and the recombinant CP assembles to *T* = 1 particles that encapsidate that mRNA, suggestive of packaging specificity [24]. We tested this idea by selecting preferred RNA binding ligands using the SELEX technique in combination with bioinformatics. This identified a short stem-loop (B3) with a single-stranded loop motif of –A.X.X.A-, where X = any nucleotide. There are multiple versions of this recognition signal, a putative PS, in all three STNV genomes [13]. We tested whether this sequence was functionally important in in vitro reassembly assays comparing a mutant sequence with a loop of –U.U.U.U- as a control [14]. The STNV CP does not assemble beyond monomer in the absence of RNA. Both B3 and the 4U variant trigger self-assembly of *T* = 1 capsids with STNV CP, but with dramatically different efficiencies, the –A.X.X.A- version being much more efficient. X-ray structure determination of the virus-like particle (VLP) created by B3-induced assembly reveals repeated RNA-CP contacts around the helices that occur at the virion three-fold axes. Compared to the virion structure, these helices have become more ordered allowing a cluster of positively charged side-chains to come into close contact. These aptamer sequences appear to overcome the potential electrostatic repulsion between CP monomers and are obvious candidates to be the PSs in the STNV RNA inferred from the smFCS experiment.

Similarly, RNA SELEX and known structures for CP-binding RNA stem-loops suggested a characteristic motif for PSs in MS2 [25–27]. Using a novel interdisciplinary approach (Dykeman et al., in preparation), combining SELEX and structure function data with graph theoretical tools (Hamiltonian paths) we were able to identify the 60 PSs consistent with the DSM, and map them into the tertiary structure of the packaged genome, i.e., we associated all PSs with defined positions in the capsid. This analysis revealed a striking conclusion: the organization of the packaged genome in contact with capsid is much more constrained than previously appreciated. This is consistent with a recent cryo-tomographic structure determination of the MS2 virion in contact with its primary cellular receptor, the bacterial pilus. Using the pilus for alignment, it has been possible to determine a tomographic structure for the bound phage without symmetry averaging of the density (Dent et al., submitted). Tomograms of individual particles have too low a signal-to-noise ratio to provide useful information but by sub-tomographic averaging of many asymmetric particles it is possible to generate an interpretable structure. This confirms that the capsid is based on icosahedral symmetry everywhere except the contact point with the pilus, where it appears that a coat protein dimer has been replaced by the single copy of maturation protein. Within the protein shell there is density that must correspond to the genomic RNA, both in contact with the protein shell, as expected from the roles of the RNA packaging signals, and at lower radii. Due to the averaging between particles, the strength of this density shows that the conformation of encapsidated RNA in every particle must be very similar. Indeed, we tested the data for correlation with the structures of any of the possible Hamiltonian path organizations and identified a single path as the best fit (Geraets et al., in preparation). Interestingly, it is the same path that had been identified earlier via an independent approach based on assembly kinetics [12] as the path describing the likely organization of genomic RNA in proximity to capsid.

A similar analysis of the PSs in an evolutionarily related phage, GA, revealed a different PS motif and different PS distributions in the secondary structure of that packaged genome, but the same contact pattern between PS in the RNA organization in proximity of the capsid, pointing to an evolutionarily conserved packaging arrangement in this family of RNA viruses.

## Packaging signals and particle geometry

The tight link between the structure of the packaged genome and capsid assembly implies a conserved assembly mechanism for these RNA viruses. This is because the positions of the PSs and the way in which the genomic RNA is organized between them define the assembly pathways and intermediates. For example, for MS2 assembly is assumed to nucleate at the highest affinity PS (TR, Fig. 1), which in this case is located approximately at the center of the genomic sequence. After this, assembly proceeds along the RNA towards the 5′ and 3′ ends simultaneously via recruitment of additional CP dimers, consistent with the experimental observation of capsid intermediates seen via mass spectrometry. In such a co-assembly scenario, formation of CP complexes is correlated with the organization of the genomic RNA in contact with it. Hence, the organization of the genome and the associated positioning of the PSs reflect the geometry of the capsid intermediates and the final virion. Therefore, mathematical tools describing capsid geometry can be used to characterize the assembly process. In particular, the concept of Hamiltonian paths, which we introduced as a tool to enumerate all possible organizations of the packaged genomes in contact with capsid [28, 29], allowed us, in combination with techniques from biochemistry and biophysics, to characterize the local rules according to which these viruses form [12]. This work has revealed a preferred assembly pathway, which is consistent both with the independent analysis of the PS distribution, and with results of the recent cryo-TM study.

## The function of packaging signals

**Fig. 3:**
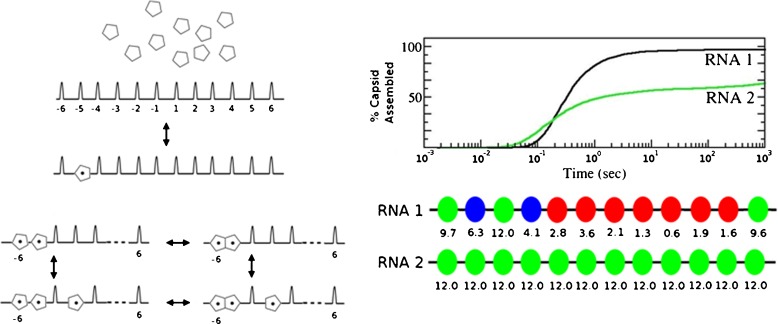
Modeling the effects of different packaging signal affinities. In order to assess the impact of different distributions of PS affinities to capsomeres, we analyze the assembly scenario given by the assembly reactions in the *left panel*. These assume nucleation at a designated high affinity signal via recruitment of a CP (*pentagon*). Further CP-RNA contacts can be formed or broken with defined on- and off-rates, and CPs bound to neighboring PSs can associate (and potentially dissociate again). The *right panel* shows the resulting particle yield depending on the strengths of the PS-CP contacts across the RNA genome (varying between 0 and −12 kcal/mol in increments of 0.1 kcal/mol), obtained via a Gillespie-type algorithm sampling the possible assembly pathways. The analysis revealed that heterogeneous PS affinities distributions performed consistently better in terms of completed capsid than scenarios in which all PS affinities are equal; an example of each is shown for illustration

These results pave the way for a better understanding of how the PSs contribute to making capsid assembly both efficient and accurate. The existence of multiple PSs suggests that they fulfil a regulatory role during capsid assembly, which is perhaps surprising, given that the largest fraction of them (e.g., over 70% in MS2 (Dykeman et al., in preparation)) have relatively weak affinity for their cognate CP. In order to understand this phenomenon, we have analyzed capsid assembly for a dodecahedral model system consisting of 12 pentagonal building blocks that can interact with 12 packaging signals on a hypothetical RNA (Fig. 2c) [30]. Assembly reactions (Fig. 3a) assume nucleation at a designated high affinity signal via recruitment of a CP (pentagon). Further CP-RNA contacts can be formed or broken with defined on- and off-rates, and CPs bound to neighboring PSs can associate (and potentially dissociate again). We analyzed the efficiency of capsid assembly in terms of particle yield depending on the strengths of the PS-CP contacts across the RNA genome using a Gillespie-type algorithm to sample the possible assembly pathways. The Gillespie algorithm uses a stochastic framework to compute the reaction kinetics of a solution of chemicals. Given a set of possible reactions, such as a ligand-binding event or the dissociation of a ligand–protein complex, the algorithm computes the probability that each reaction would occur within a time increment and picks one reaction to “fire” based on these probabilities. Our algorithm for capsid assembly in the presence of RNA uses the algorithm to compute the reaction kinetics for a solution of CP and RNAs that contain multiple PSs with varying affinities for CP [30]. The reaction probabilities are estimated from the forward and backwards reaction rates, which satisfy the equilibrium equation:

$$ \frac{k_f }{k_b }=e^{-{\Delta}{G}/RT} $$

where R is the gas constant and T is the temperature (here chosen as 298 K).

The analysis revealed that heterogeneous PS affinity distributions (in this model of CP-RNA affinities between 0 and −12 kcal/mol) performed consistently better than RNAs with identical PSs, such as homogeneous polymers (i.e., the situation when all PS affinities are equal). An example of each scenario is shown in Fig. 3b. An analysis of the assembly pathways of better performing RNAs revealed that PSs of weak affinity are located predominantly in positions where dissociation may be important for error correction on the pathways, while strong packaging signals mark positions that do not require dissociation to complete capsid formation. This suggests an intimate link between capsid geometry and assembly kinetics that is mediated via the affinities of the PSs. Ongoing work suggests that the PS are evolutionarily tunable (Dykeman et al., in preparation), like knobs on a radio, adapting the RNA to capsid geometry so as to optimize capsid yield during assembly.

## Conclusions

Inspired by the remarkable insights into RNA virus assembly provided by the single-molecule experiments for MS2 and STNV [6], we have developed a new model for the capsid-genome co-assembly process in ssRNA viruses. A central element of this approach is the packaging signal hypothesis, which suggests that repeated contacts between RNA and CP, mediated by the PS, have a regulatory role in capsid assembly, hence making this process more efficient. This regulatory role can manifest itself in different ways, e.g., via allosteric dimer switching as in MS2, or overcoming of electrostatic barriers in capsid protein association as in STNV. We have shown that the affinities of PS to capsid protein are key in adapting the RNA for efficient packaging into a capsid of a defined geometry, making the genomic RNA a finely tuned molecular machine primed to optimize capsid assembly. This is vital given that capsid efficiency is important in each viral particle’s race against its host’s natural defense mechanisms.

Our analysis has shown that electrostatics alone does not account for the packaging mechanism in ssRNA viruses as it overlooks these subtle, yet vital, effects. Indeed, in vitro studies at unnaturally high CP concentrations mask this effect, which is perhaps why it had not been discovered previously. For instance, in the smFCS assays of STNV assembly at low CP concentrations (100 nM), there is clear discrimination in favor of the cognate CP-genomic RNA complex against MS2 genomic RNA and its fragments, and *vice versa* (Fig. 1). However, MS2 PSs also have loop sequences that match those of STNV (-A.U.U.A- vs. –A.X.X.A). The relative locations of these PSs in the respective RNAs are, however, optimized to promote only the cognate CP-CP contacts required to form the appropriate capsid, hence preventing assembly with non-cognate RNAs at low concentration [31]. This effect is reduced when we compare discrimination of MS2 and STNV RNAs at typical in vitro reassembly concentrations (≥10 $\upmu $M) [14]. As our modeling has shown, capsids can be formed around any negatively charged polymers as long as they fit into the volume of the capsid; however, for non-cognate RNAs, the efficiency of this process is much reduced. Our approach is a vehicle to quantify this, and for relating PS affinities with capsid yield. It is therefore essential for understanding virus assembly in vivo. We have shown that under comparable conditions (solution conditions and assumptions on on/off-rates), heterogeneous distributions of PS affinities perform consistently better, and that there is at least a difference of 5% in capsid yield with regards to the best performing polymer with a homogeneous PS distribution. This difference, albeit small, is important in an evolutionary context, as a population of viruses would out-compete another over which it has a 5% advantage in yield in only a couple of generation cycles.

These insights suggest a novel approach to anti-viral drug design, i.e., targeting the formation of the vital PS-CP contacts, and/or their consequences. Indeed, we have shown that blocking the CP-induced collapse of MS2 RNA ablates capsid assembly and others have demonstrated that a clinically approved alkaloid that binds to extra helical bases in RNA blocks assembly of TMV in vitro from its known PS. The packaging signal hypothesis may hence pave the way to a novel antiviral therapy.
